# Evaluation of the Concurrent Validity of the Oral Health Screening Tool (OHS) in the Mental Health Care Setting: A Pilot Study

**DOI:** 10.1111/scd.70165

**Published:** 2026-04-08

**Authors:** Evandro Siqueira Pires, Johanna de Almeida Mello, Joke Duyck, Elton Ferlin, Alice Hirdes, John Hirdes, Pedro Antonio Gonzales Hernandez

**Affiliations:** ^1^ Departamento de Odontologia Universidade Luterana do Brasil Porto Alegre Brazil; ^2^ Department of Oral Health Sciences KU LEUVEN Leuven Belgium; ^3^ Diretoria de Pesquisa Hospital De Clínicas de Porto Alegre Porto Alegre Brazil; ^4^ Pós‐Graduação em Enfermagem Universidade Federal de Rio Grande Rio Grande Brazil; ^5^ School of Public Health Sciences University of Waterloo Waterloo Canada

## Abstract

**Aims:**

The prevalence of oral health problems in people with mental health disorders and addictions is significantly higher than in the general population. Timely and regular assessment of their oral health is therefore important to prevent or stop their oral health deterioration. The aim of this study is to evaluate whether the Oral Health Screener for use within interRAI (OHS) accurately detects oral health care needs people in the mental health care setting in Brazil compared to the gold standard instrument for assessing oral health, the SB BRASIL 2020.

**Methods and Results:**

This is a cross‐sectional pilot study performed at the Mental Health unit of the University Hospital of the University Hospital of Canoas, Rio Grande do Sul, in the South of Brazil. Admitted patients with severe mental disorders and addictions were included, according to the inclusion criteria. First the OHS was translated and adapted to the Brazilian mental health care context. Secondly, the patients’ oral health was screened by a dentist using the OHS as well as the SB BRASIL 2020. Scores of the OHS were compared with the scores of selected corresponding items of the SB BRASIL 2020 to calculate sensitivity, specificity, predictive validity and ROC analysis of the OHS.

A total of 44 participants were included (mean age 37.5 ± 14.2, 54.6% female), of which 39% had addiction problems, 16% had bipolar disorder, and 14% depression disorder as their primary diagnosis. The items' pain and oral hygiene showed large predictive accuracy with respectively 0.82 ±0.06 and 0.85 ± 0.08. The pain item showed the best results for sensitivity (90.9%), followed by the item oral hygiene (89.47%), and condition of the teeth (75.8%). No conclusive results were found for the items palate and tongue problems, as these conditions were barely present in the sample.

**Conclusion:**

The OHS presented high sensitivity and specificity and the application of this tool in the mental health care setting can bring an added value to clinical practice, as it is much shorter and hence more feasible to be used in the daily care for people with mental disorders and addictions. A valid screening tool for oral health in the mental health care setting can help caregivers detect people with oral disease or at risk for oral health problems. This allows targeted preventive measures, such as better oral hygiene practices and referrals to dental professionals.

## 1 Introduction

The maintenance of adequate oral health plays an essential role in general health and is linked to physical health, wellbeing and social interactions. In addition, it is recognized that oral diseases can impact a person's health and quality of life [1, 2]. Daily oral hygiene is key for a healthy oral cavity and a good daily oral care is even more important for people with vulnerable health or who are care‐dependent, as they have limited abilities to perform their own hygiene [3, 4]. People with mental illnesses and addictions fall under this category, as they perceive difficulties in caring for themselves, especially when under the influence of addictive substances or in crisis situations [5, 6]. In addition, they often have more difficulties accessing care, due to financial constraints, stigma, housing instability, among other factors. In turn, deteriorated oral health compromises resocialization, which can increase the risk of unemployment, social isolation, and poverty [7, 8]. Studies have shown that patients with mental disorders and addictions, due to their psychopathology, perceive a lack of motivation in relation to oral health [9]. They often consume more cariogenic substances than the general population, either through food or as constituents of psychotropic medications; and smoke or consume alcohol more often [5, 10, 11]. Moreover, rejection and phobia in relation to dental treatment are associated with oral alterations which are directly associated with the length of drug use and exposure [12, 13, 14, 15].

In general, results from clinical and interventional studies show that dental treatments can improve the quality of life of those treated, although working on prevention of oral problems is usually less costly and can be effective [16, 17]. In the dental practice, there are instruments to detect oral health deterioration, but there are fewer instruments to routinely collect information on oral health conditions in other care practices such as nursing homes, hospitals, and so on. In addition, these are not yet applied in the mental health care settings [18, 19]. As people with mental health disorders and addictions often do not have contact with a dental professional, it is the caregivers involved in their daily care who could help detect their oral health problems. In order to establish a practical, concise and effective tool for oral health assessment, a screening tool was developed for non‐dental professionals: the Oral Health Screener for use within interRAI, also called the OHS.

The interRAI tools are comprehensive assessment instruments, standardized and validated for different care settings, including several domains such as cognitive functioning, psychosocial situation, health status, diagnoses, and environmental aspects. In addition, the instruments include a section about oral health, the OHS, which makes it possible to link oral health situations with general health [20, 21]. A systematic review by Rodrigues et al. (2023) analyzed several oral health assessment instruments and concluded that this tool was considered the most suitable instrument for non‐dental caregivers [22].

In the OHS tool, the oral health screening is carried out by non‐dental health professionals. Although the OHS was developed for use in the home care setting or in nursing homes, other care settings might also benefit from this assessment. By timely and regularly assessing the oral health of people with mental health disorders and addictions, preventive measures can be taken such as providing better oral hygiene to these patients and referring them to a dental professional, when necessary. In addition, the assessment itself can bring awareness to caregivers on the importance of oral health. The aim of this study is to evaluate the concurrent validity of the OHS to be used as an oral health screening instrument for people with mental health illnesses.

## 2 Materials and Methods

This is a cross‐ sectional non‐randomized study carried out in the Mental Health Unit of the University Hospital of of the University Hospital of Canoas, Rio Grande do Sul, in the South of Brazil. The study population consists of patients with severe mental disorders and/or addictions, admitted to the Mental Health Inpatient Units (juvenile and adult) of the hospital. The dental treatment is offered at the Dentistry department based at the aforementioned University Hospital.

### 2.1 Consents

The study was approved by the Human Research Ethics Committee (CEP) of the Universidade Luterana do Brasil (ULBRA), No. 5.434.490/2022 linked to the research project “Oral health as a predictive variable in the quality of life of people with mental disorders and addictions”. Permission was obtained from the research group Population Studies in Oral Health (PSIOH), in the Department of Oral Health Sciences of the KU Leuven (Leuven, Belgium) and from the interRAI consortium (http://www.interrai.org) to translate the OHS‐interRAI into the Brazilian Portuguese language and to test the instrument in the mental health care setting. Written informed consents were obtained from all participants.

### 2.2 Participants

Participants were recruited for the testing of the OHS at their admission to the Mental Health unit of the University Hospital of Canoas. People over 18 years old with severe mental disorders and/or addictions were included. Patients in acute condition (psychosis) or with moderate or severe cognitive impairment were excluded from the study, according to the psychiatric medical diagnosis drawn up by the attending professional, and referenced in the corresponding hospital records. Patients in need of total or partial dental prostheses or orthodontic treatment were excluded from the study. This decision was based on the limited duration of the pilot study (10 months), and on the clinical characteristics of the study population, composed of individuals with mental disorders and substance use disorders. These patients usually present crisis episodes and have significant difficulties in undergoing and adhering to more complex dental treatments, such as orthodontic or prosthetic care. Due to feasibility, these patients had to be excluded.

### 2.3 Study Preparation

Before the start of the study, the OHS instrument was translated into Brazilian Portuguese and adapted to the mental health care setting, considering cultural, idiomatic, linguistic and contextual aspects [23, 24]. The first requirement for cross‐cultural comparisons of instruments is conceptual/functional equivalence which, rather than direct translation and comparison of instruments, is achieved through translation and back‐translation [25, 26]. One example of an oral health assessment which has been translated into several languages is the OHIP‐14. This instrument measures the impact of oral health in someone's life, which can be an indication of oral health quality of life. The OHIP‐14 instrument has its original version in English and it was translated into several languages, including Brazilian Portuguese [27, 28]. This offers the possibility of making international comparisons. The OHS was created in Dutch and is currently available in English, French and German.

The translation process of the OHS into Brazilian Portuguese followed the flow and criteria suggested by Beaton et al. [26]. The construct was first submitted to two sworn professional translators, native speakers of Brazilian Portuguese. The translations were then sent to a committee of experts made up of two researchers with expertise in dentistry and proficiency in Portuguese and English, who took the role of judges [29]. The committee drafted the synthesis version (or final version by consensus) and forwarded it for back‐translation by a native translator. The back‐translated text was analyzed again by the experts, who checked it against the original document, thus achieving a final agreement level of 99% [25, 30, 31].

### 2.4 Instruments

The OHS consists of nine items to assess oral health (acceptable/unacceptable). The items chewing function, discomfort or pain and dry mouth are assessed using specific questions answered by the patient. The other six items hygiene of removable prostheses, oral hygiene, teeth, gums, tongue and palate, and the inner surface of the cheeks and lips require visual inspection. Photographs are provided to support the assessment [20, 21].

The SB Brasil 2020 is the gold standard instrument for assessing oral health and is used in dental consultations, as well as in the National Survey of Oral Health in Brazil [32, 33]. It is a comprehensive instrument to be completed through a dental examination conducted by a qualified dentist. Full equipment is necessary, including specific extraoral and intraoral light sources to examine all oral structures, as well as hard and soft tissues [34]. In this research, the most recent version of the instrument was used: the SB BRASIL 2020, which can be found in the Supplementary Material. An additional questionnaire was created to ask for socio‐demographic characteristics of the patients, as well as a question about their diagnosis.

### 2.5 Data Collection

First, items from the SB BRASIL 2020 (gold standard) were selected which could be compared with items from the OHS. The selection was made by dental experts from the research teams of the of the ULBRA and the KU Leuven universities. The items were dichotomized to indicate two outcomes: healthy or unhealthy condition. Six of the nine items of the OHS had similar items in the SB BRASIL 2020: pain, teeth, oral hygiene, gums, tongue, and soft tissues (lips and palate). The other items of the OHS (chewing function, dry mouth, and hygiene of the prosthesis) were all assessed, but no direct correspondence was found in the SB BRASIL 2020. Specifically, the SB BRASIL 2020 does not include questions on oral function such as chewing, and it does not collect information on xerostomia or dry mouth and on the hygiene of dental prostheses. Therefore, validation for these three items was not possible. This reflects differences in the scope and objectives of the two instruments rather than a weakness, as these items capture relevant clinical dimensions of oral health that are not addressed in population‐based surveillance surveys such as the SB BRASIL 2020.

To standardize criteria, minimize error variability, and correct discrepancies in instrument completion among examiners, a comprehensive training and calibration process was conducted. This process aimed to organize and address potential technical and operational issues, assess comprehension of questionnaire items, estimate the time required for data collection, and calibrate the team of examiners. Two experienced dentists were designated as gold‐standard examiners. These calibrators conducted assessments in 10 individuals, who were also independently evaluated by each member of the survey team, following established guidelines. Results were compared, and in cases of substantial discrepancies, participants were re‐examined to allow inter‐examiner differences to be reviewed and resolved through group discussion. The study was initiated only after achieving a Kappa agreement coefficient of at least 0.81 for each item.

Dentists from the dental clinic of the hospital assessed the oral health of the participants first using the OHS and then using the SB BRASIL 2020. This order of assessment was fixed, as the SB BRASIL requires the use of a dental mirror and a probe, which could bias the OHS results, as the latter does not require any equipment. The following items were assessed in both instruments:

- Pain in the OHS: presence of pain or discomfort (yes/no). Pain in the SB BRASIL 2020: item found in the identified emergencies category, sub‐item pain of dental origin.
- Gums in the OHS: Look healthy/not healthy. Gums in the SB BRASIL2020: included under periodontal conditions, sub‐item bleeding on probing.
- Teeth in the OHS: intact teeth/not intact. Teeth in the SB BRASIL 2020: included under the dental examination category, sub‐items conditions per tooth.
- Oral hygiene in the OHS: acceptable/not acceptable. Oral hygiene in the SB BRASIL 2020: included under the periodontal condition category, sub‐items bleeding on probing and presence of calculus.
- Tongue in the OHS: looks intact and uniform (yes/no). Tongue in the SB BRASIL2020 included under the stomatological diagnosis category, which assesses the presence of primary lesions.
- Palate, lips and cheeks in the OHS: looks intact and uniform (yes/no). Palate, lips and cheeks in the SB BRASIL: included under the stomatological diagnosis category, which assesses the presence of primary lesions.

After a dentist assessed the oral health condition of the participant, the whole dentist team drew a dental treatment plan for the participant and carried out the plan during the months following the first assessment.

### 2.6 Statistical Analysis

First descriptive statistics were performed for the main characteristics of the participants. Then responses to the items of the OHS and the SB BRASIL 2020 were illustrated with contingence tables. To evaluate the performance of the OHS assessment compared to the SB BRASIL 2020, a Receiver Operating Characteristic curve (ROC) analysis was performed, as it is known as an effective method for evaluating the performance of an instrument or test [35, 36]. The Area Under the Curve (AUC) is an overall summary of diagnostic accuracy.

## 3 Results

Table 1 shows the characteristics of the sample. A total of 44 participants were assessed after being admitted to the mental health unit. Most of them were female (54.6%) and the mean age of the sample was 37.5 (SD: ± 14.2). About 71% of the participants had a low income, earning a wage lower or equal to the Brazilian minimum salary. Regarding their mental health pathologies, the majority of participants had addiction problems (38.6%) as main diagnosis.

**TABLE 1:** Characteristics of the sample.

Characteristics (%)	*n* = 44 Mean age: 37.5 ± 14.19 (17–73)
Gender (female)	54.55%
Low income (lower or equal Brazilian minimum wage)^a^	71.43%
Mental health pathologies	
Addiction	38.63%
Bipolar disorder	15.91%
Depression	13.64%
Schizophrenia	11.36%
Other mental disorders	20.46%

^a^ Brazilian minimum wage is 1450 reais, corresponding to 272 US dollars on October 10, 2025.

The contingency table below (Table 2) shows that most proportions per oral health item were situated in the diagonals, meaning accurate results (true positives and true negatives). These were the correctly classified cases when comparing the OHS results with the SB BRASIL 2020. For the item tongue, and palate/ lips, most cases were true negatives, representing a case of class skewness, also referred to as class imbalance, indicating that for these items the negative true cases outnumber the positive true cases [37].

**TABLE 2:** Contingency tables for each evaluated item.

OHS‐items	SB BRASIL 2020 diagnostic	
Pain	Pain (Yes)	Pain (No)
Yes	22.7% (TP^a^)	20.4% (FP^b^)
No	2.3% (FN^c^)	54.6% (TN^d^)
Gums	Gum (Yes)	Gum (No)
Yes	15.4% (TP)	12.8% (FP)
No	15.4% (FN)	56.4% (TN)
Tooth	Tooth (Yes)	Tooth (No)
Yes	62.5% (TP)	2.5% (FP)
No	20.0% (FN)	15.0% (TN)
Oral hygiene (teeth)	Oral hygiene (Yes)	Oral hygiene (No)
Yes	58.6% (TP)	6.9% (FP)
No	6.9% (FN)	27.6% (TN)
Tongue	Tongue (Yes)	Tongue (No)
Yes	0% (TP)	2.4% (FP)
No	2.4% (FN)	95.2% (TN)
Palate/lips	Palate/lips (Yes)	Palate/lips (No)
Yes	0% (TP)	4.7% (FP)
No	2.4% (FN)	92.9% (TN)

^a^ TP: True Positives.

^b^ FP: False Positives.

^c^ FN: False Negatives.

^d^ TN: True Negatives.

**FIGURE 1:**
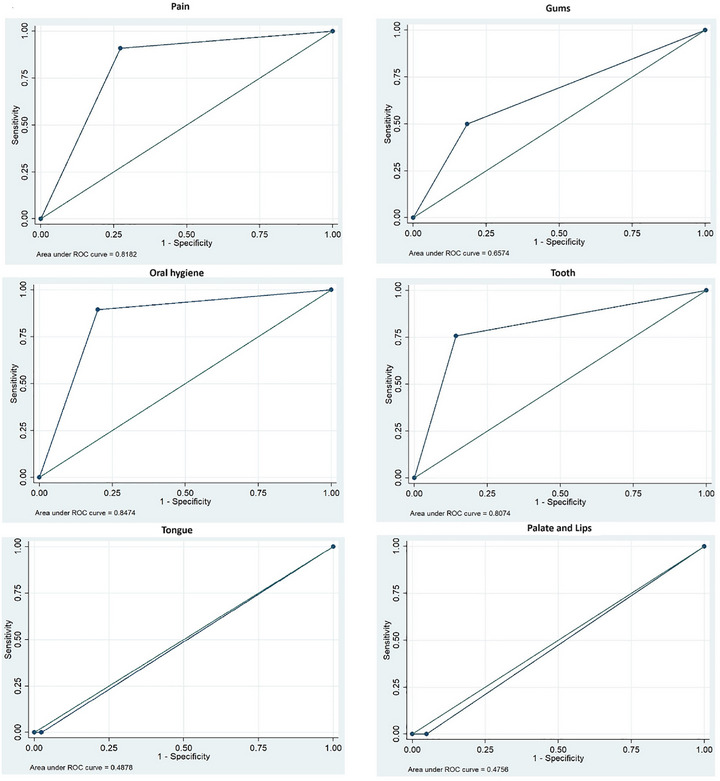
Receiver operating characteristic curve (ROC) plots for the items of the OHS instrument in relation with the SB BRASIL 2020.

Table 3 and Figure 1 show the results for the ROC analysis for the six items of the OHS and the SB BRASIL 2020. Results showed large predictive accuracy for the items pain and oral hygiene, respectively 0.82 and 0.85, as well as high sensitivity (90.9% and 89.47%). The item oral hygiene showed 80.0% specificity, and 86.2% correct classification of cases. The item tooth showed 75.7% sensitivity, 85.7% specificity, and 77.5% correct classification of cases, with a predictive accuracy of 0.81. Although the item gum showed high specificity of 81.4%, a low sensitivity rate was found of 50.0%, as well as a correct classification of 71.7% and lower predictive accuracy of 0.66. This finding is attributable to the lack of comparable constructs in the gold standard rather than to poor performance of the OHS items themselves.

**TABLE 3:** Characteristics of the Receiver Operating Characteristic curve (ROC) for the OHS instrument in relation with the SB BRASIL 2020.

Variable	ROC	Sensitivity	Specificity	Correctly classified
Pain	0.82 ± 0.06	90.91%	72.73%	77.27%
Oral hygiene (teeth)	0.85 ± 0.08	89.47%	80.00%	86.21%
Teeth	0.81 ± 0.08	75.76%	85.71%	77.50%
Gums	0.66 ± 0.08	50.00%	81.48%	71.79%
Tongue	—	—	97.56%	95.24%
Palate and lips	—	—	95.12%	92.86%

The item tongue and the item palate/lips presented class skewness, as most of the cases were true negatives, with most values as 0 (outcome not present). Therefore, sensitivity could not be calculated, but the items showed high specificity (97.56% and 95.12 respectively) and higher correct classification (95.24% and 92.8%) than the other items, although this correct classification is only for the true negatives. For both items, the ROC curves were below the 45° diagonal line, with no conclusive results. As the correct classification was driven almost exclusively by true negatives, these metrics do not provide information about the ability of the items to detect true positive cases, as they were merely present in the sample. Consequently, the ROC curves fell below the 45° diagonal line. This indicates limited discriminative capacity in this context, definitive conclusions regarding concurrent criterion validity for these items.

## 4 Discussion

This study evaluated the concurrent validity of the OHS tool, comparing it to the well‐established diagnostic instrument SB BRASIL 2020. This is the first study to apply the OHS to the setting of mental health care and addictions and to test the OHS tool in Brazil.

The study sample was composed mainly of young adults with mental illnesses and/or addictions, with an income lower than the Brazilian minimum wage. This profile is consistent with literature where significant associations are found between mental health disease, unemployment rates and economic inactivity in Brazil [38].

The variables pain, oral hygiene and teeth showed the highest sensitivity and specificity, when compared to the corresponding diagnostic items of the SB BRASIL 2020. This is similar to the study of Krausch‐Hofmann et al. where a high specificity rate was found for the item pain and high sensitivity for the item teeth [7]. The item pain in the SB BRASIL 2020 is more detailed than in the OHS, as it indicates not only the presence of pain but also the source of the pain (e.g. acute abscess, chronic abscess, etc.). The item gums yielded the lowest results for predictive validity and sensitivity, but high specificity, consistent with the study of Krausch‐Hofmann et al. [39]. In the OHS, only the appearance of the gums is assessed. Through the use of the OHS, it is possible to identify gum problems and to improve oral hygiene, without making a diagnosis of oral disease [21]. However, as the SB BRASIL 2020 is a diagnostic tool, the assessment is carried out using an instrument to assess the diagnosis of gingivitis, a probe, evaluating the presence of gingival bleeding, gingival retraction and/or pockets, which are factors for considering periodontal disease, although the gum items had the lowest rates of correct classification and predictive accuracy [34].

A study by Midwood et al. (2019) assessed patients' periodontal health self‐awareness using a questionnaire, followed by a clinical assessment of periodontal probing depth, gingival recession, the presence of gingival bleeding and/or exposed dentin [40]. As a result, there was general agreement between the clinical assessment and the participants' self‐reported tooth mobility and gingival recession, but a low sensitivity and specificity for gingival bleeding, as it tended to be underestimated by participants. Baudet et al. (2020) found that 82.5% of people in their study considered gingival bleeding to be something natural and not associated with gingivitis. This showed the lack of knowledge about periodontal diseases among the population and pointed to the importance of oral hygiene instructions [41]. The gum item of the OHS only assesses the color and aspect of the gums and does not cause bleeding, as the tool is to be assessed by non‐dental professionals only by visual inspection in the mouth. Visual inspection is considered highly effective and reliable for oral examination [42]. It is effective for detecting changes in texture of the gums, loss of surface integrity, as well as identifying parameters such as color and size of lesions [43].

The palate and tongue items did not present a conclusive ROC curve due to the absence of enough cases with a true positive rate (class skewness). However, both items presented similarly high results for specificity, showing consistent results with Krausch‐Hofmann et al. [39]. To analyze palate and tongue conditions, studies often focus on determining sensitivity and specificity through a variety of assessment methods, including clinical examinations, imaging techniques, as well as molecular testing. If the prevalence is sufficient, a positive result tends to confirm the presence of the disease, while if the prevalence is low, a positive result does not allow its confirmation [44]. As mental illnesses or addictions are known to be associated with higher risk for oral squamous cell cancers, as well as neck and head cancers, the inability to detect or accurately record abnormalities in these soft regions could delay referral for definitive diagnosis [45, 46]. To mitigate this risk, the OHS emphasizes the identification of ulcers, color alterations, swelling, bleeding, or non‐healing lesions, which are commonly associated with clinically significant pathology. In addition, the use of the OHS should be accompanied by clear referral pathways to dental or specialized care whenever abnormalities are suspected. These strategies may help increase sensitivity while preserving the feasibility of the instrument in mental health care settings. In this study we could not conclusively validate these items, but literature recommends to perform tests of sensitivity for rare/not sufficient positive cases using a targeted population with a high risk for these cases [47].

The present study showed that the OHS can be used in the mental health care setting as a tool to detect care needs and hence refer the patient to dental treatment. An advantage of the OHS is that it can be used by non‐dental professionals, whereas this is not the case with the SB BRASIL 2020, being a diagnostic tool. Further studies should test the OHS in the mental health care setting at a larger scale.

### 4.1 Strengths and Limitations

Concurrent criterion validity was assessed by dentists, as the reference instrument SB BRASIL 2020 requires clinical expertise in dentistry and cannot be applied by non‐dental caregivers. Although the OHS is intended for use by non‐dental caregivers, its validation against a gold standard necessarily involved trained dental professionals. Further validation studies involving non‐dental caregivers are planned and are essential to confirm the applicability of the OHS in routine mental health care settings, which is the primary purpose of the instrument. This will be done in the future with instruments such as the OHIP or the ROAG, which are designed for non‐dental caregivers.

As this was a pilot study to validate the OHS instrument in the mental health care setting in Brazil, a small sample size was used. Each patient received dental treatment, so it was not feasible to include more patients, due to financial and time constraints. Although the sample size was small, a diverse sample of participants was included with varying age, income, as well as mental health diagnosis. Due to feasibility, patients in need of prostheses or orthodontic treatment were not included, which may have biased the sample.

The main strength of the research is the use of the SB BRASIL 2020 as the benchmark, which is an instrument well‐established in Brazil and very detailed, with a high diagnostic accuracy, which reduces measurement error bias. The fact that the SB BRASIL 2020 does not include questions on oral function such as chewing, does not collect information on xerostomia or dry mouth, and does not assess the use or hygiene of dental prostheses, hindered validation of these three items of the OHS. As the SB BRASIL 2020 is a gold standard used in population‐based surveillance surveys, further validation for these three items of the OHS will be necessary. The lack of these items highlights that the OHS captures clinically meaningful aspects of oral health that extend beyond the scope of large epidemiological surveys. This strengthens the argument that the inability to validate this item reflects a gap in the gold standard, not a lack of importance or validity of the construct being measured.

Overall, the findings demonstrate that the OHS has a preliminary level of validity in mental health care settings and that generalization should be undertaken with care. Future studies should include larger, more heterogeneous samples, including individuals with prosthetic or orthodontic needs and patients in other mental health care settings, such as inpatient mental health and outpatient. Validation by non‐dental caregivers is essential to evaluate real‐world applicability and ensure effective referral for identified oral health issues. Increasing both sample size and broadening the settings will improve external validity and provide clearer evidence of the potential of the OHS to improve detection of oral health conditions, monitoring and oral care for people with mental health illnesses.

## 5 Conclusion

The items of the OHS performed well on concurrent validity when compared with the diagnostic tool SB BRASIL 2020. The tool can be used to detect oral health problems timely and to refer patients to dental treatment. The application of the OHS in the mental health care setting can bring an added value to clinical practice, as it is known that oral diseases are very prevalent in people with mental disorders and addictions.

## Funding

The research was funded by the Coordenação de Aperfeiçoamento de Pessoal de Nível Superior (CAPES).

## Conflicts of Interest

The authors declare no conflicts of interest.

## Data Availability

Data used in this study is available upon reasonable request.
